# Grafting Delays Watermel on Fruit Ripening by Altering Gene Expression of ABA Centric Phytohormone Signaling

**DOI:** 10.3389/fpls.2021.624319

**Published:** 2021-02-25

**Authors:** Shaogui Guo, Honghe Sun, Jiaxing Tian, Guoyu Zhang, Guoyi Gong, Yi Ren, Jie Zhang, Maoying Li, Haiying Zhang, Haizhen Li, Yong Xu

**Affiliations:** National Watermelon and Melon Improvement Center, Key Laboratory of Biology and Genetic Improvement of Horticultural Crops (North China), Beijing Key Laboratory of Vegetable Germplasm Improvement, National Engineering Research Center for Vegetables, Beijing Academy of Agriculture and Forestry Sciences, Beijing, China

**Keywords:** watermelon, graft, fruit development, gene expression, phytohormone

## Abstract

Grafting cultivation is implemented worldwide mainly to resist abiotic and biotic stresses and is an effective method to improve watermelon production. However, grafting may affect fruit development and quality. In our experiment, pumpkin-grafted (PG) watermelon fruits developed slower and the ripening period was extended compared to self-grafted (SG) fruits. We found that the concentrations of abscisic acid (ABA) among endogenous phytohormones were dramatically reduced by pumpkin grafting. In order to understand these changes at the gene expression level, we performed a comprehensive analysis of the fruit flesh transcriptomes between PG and SG during fruit development and ripening. A total of 1,675 and 4,102 differentially expressed genes (DEGs) were identified between PG and SG. Further functional enrichment analysis revealed that these DEGs were associated with carbohydrate biosynthesis, phytohormone signaling transmission, and cell wall metabolism categories. ABA centric phytohormone signaling and fruit quality-related genes including ABA receptor, PP2C proteins, AP2-EREBP transcription factors, sucrose transporter, and carotenoid isomerase were co-expressed with fruit ripening. These results provide the valuable resource for understanding the mechanism of pumpkin grafting effect on watermelon fruit ripening and quality development.

## Introduction

Watermelon [*Citrullus lanatus* (Thunb.) Matsum. & Nakai var. *lanatus*], belonging to the Cucurbitaceae family, is an important global horticultural crop species. Watermelon growing area has been continuously expanding. Steel-frame sheds, greenhouses, and other protected areas cause severe spreading of soilborne diseases. Severe Fusarium wilt outbreaks can result in the destruction of whole croplands. Although chemical control agents, biological control agents, and physical prevention and control technologies have been used to attempt to solve this problem, there is no effective control measure. Grafting is a useful cultivation technology initially applied in fruit tree planting. Now it is widely employed in horticultural plant production. Watermelon grafting has been utilized in China since the 1960s and was accepted gradually by more countries and areas. The application of grafting in watermelon crops has been used to solve major problems associated with Fusarium wilt. Grafting was used to enhance the growth vigor and soilborne resistance, extend the fruit development period, and then promote fruit yield. The mechanism of grafting cultivation-induced improvement in plant growth and fruit development becomes the research focus recently. Several studies have been carried out to dissect the grafting-induced changes in phenotypes, physiology, compositions, gene expressions, and signal transmission in watermelon, cucumber, melon, and tomato (Fredes et al., 2017; Aslam et al., 2020; Fallik and Ziv, 2020; Garcia-Lozano et al., 2020; Chen et al., 2021). In general, the yield and the fruit quality of the scion were affected by the rootstock (Davis et al., 2008). Fruit of the *Cucurbita* rootstock-grafted watermelon shows the firm and rough flesh texture (Kawaide, 1985). Grafting cultivation reduces the flesh sweetness during watermelon fruit development (Qian et al., 2004). Liu et al. (2016) reported that the lycopene contents were different in the flesh of pumpkin-grafted (PG) watermelon fruit and self-grafted (SG) watermelon fruit. In total, 14 differentially expressed genes (DEGs) from 8 gene families were suggested in mediating lycopene biosynthesis in the fruit of the grafted watermelon. Besides flesh sweetness, color, and texture, other watermelon fruit traits including fruit shape and rind thickness were also affected by grafting cultivation (Yetisir et al., 2003). Kong et al. (2016) found that watermelon grafting cultivation using bottle gourd and wild watermelon increased fruit lycopene content. At the transcriptome level, the biosynthetic genes, ζ-carotene desaturase, and phytoene synthase, were upregulated. In contrast, the catabolic genes, carotenoid cleavage dioxygenase, beta-carotene hydroxylase, 9-cis-epoxycarotenoid dioxygenase, and zeaxanthin epoxidase, were downregulated. Watermelon fruit quality, yield, plant growth, and disease resistance were affected by specific rootstock grafting cultivation. The content of fruit lycopene was significantly increased by grafting (Alan et al., 2007; Proietti et al., 2008; Mohamed et al., 2012). Petropoulos et al. (2014) found that fruit volatiles concentrations in grafted watermelon were higher than those in ungrafted watermelon. Compared with the ungrafted watermelon, plant growth, fruit number, yield, and fruit flesh firmness were increased by grafting cultivation. Soteriou et al. (2014) reported that watermelon fruit ripening was delayed comprehensively by grafting cultivation. Interestingly, fruit quality of the grafted watermelon was improved finally by the extended development period. Fruit flesh firmness, lycopene content, and fruit juice titratable acidity were increased by grafting cultivation, while the dynamic peaks were delayed. Tripodi et al. (2020) found that the volatile aroma compounds of watermelon fruits were also affected by different grafting combinations. However, the impact and mechanism of grafting-induced watermelon fruit quality improvement were not well-elucidated (Nawaz et al., 2016).

To explore the material basis, gene expression patterns and the co-expressed networks that play regulatory roles in grafted watermelon during fruit ripening, high-throughput Illumina RNA sequencing (RNA-Seq) analysis, combined with the physiologic analysis and metabolic analysis, was applied to dissect the grafting-induced impacts during watermelon fruit development and ripening. The large-scale, multi-strategies dataset provides a valuable resource for understanding the mechanism of pumpkin grafting effect on watermelon fruit ripening and quality development.

## Materials and Methods

### Plant Materials and Morphological Trait Measurement

Cultivated watermelon 97103 (*Citrullus lanatus* subsp. *vulgaris*) was used as scion. Jingxinzhen No. 2 (*C. maxima* × *C. moschata*) was used as rootstock. Watermelon 97103 plants that were grafted onto watermelon 97103 were used as controls. Grafted plants were grown in plastic pots containing mixture (peat:sand:pumice, 1:1:1, v/v/v) in the greenhouse of Beijing Academy of Agriculture and Forestry Sciences. The scions at the two-leaf stage and the rootstocks at the cotyledon flattening stage were used for grafting. These pumpkin graft combinations and watermelon self-graft combinations were grown under the same conditions. Flowers were hand-pollinated and tagged. Center flesh samples at the critical stages from PG plants and from SG plants at the same stages were collected for RNA extraction and transcriptome analysis. SG fruit samples were collected at 10, 18, 26, and 34 days after pollination (DAP). PG fruit samples were collected at 10, 18, 26, 34, and 40 DAP. Three plants in each replicate and three biological replicates were used. Tissues were frozen in liquid nitrogen immediately and stored at -80°C until use.

Soluble solid content of the fruit flesh was determined by the handheld digital refractometer (PR-1; Atago, Tokyo, Japan). Each treatment was repeated three times. Each replicate consisted of five fruit samples. Frozen samples from each development stage were ground to juice by Bio-Gen PRO200 homogenizer (PRO Scientific, Oxford, United Kingdom). Lycopene was analyzed using high-performance liquid chromatography instrument (Waters, Milford, MA, United States) with a Waters PDA detector 2535 and Agilent LCZORBAXSB-C18 column (250 mm × 4.6 mm, 5 μm; Agilent), and the eluted derivative was detected at 472 nm. Lycopene authentic standard (Sigma, St. Louis, United States) was used to construct the standard curve and quantify the samples. Concentration of fruit flesh lycopene was determined as μg/g of fresh weight (FW).

Phytohormones were analyzed in the mass spectrometry (MS) laboratory of the College of Biological Sciences, China Agricultural University. Flesh samples were harvested and frozen in liquid nitrogen then stored at -80°C. Phytohormones were extracted following the description of Wang et al. (2017). Chromatographic separation was carried out on the Waters ACQUITY UPLC I-Class system (Waters Corporation, Milford, MA, United States) using Poreshell EC-120 chromatographic column (3.0 mm × 100 mm, 3 μm; Agilent, Santa Clara, CA). Mass spectrometric analyses were carried out using Thermo Q-Exactive high-resolution mass spectrometer (Thermo Scientific, Waltham, MA, United States) and the following conditions: ion source, HESI; spray voltage (–), 3,000; capillary temperature, 320; sheath gas, 30; aux gas, 10; spare gas, 5; probe heater temperature, 350; S-Lens RF level, 55. The retention time and mass spectrometry information were determined with standard substance.

### RNA Extraction and Library Preparation

Total RNA was isolated using Quick RNA Isolation Kit (Cat. No.: ZH120, Huayueyang Biotechnology, Beijing, China) following the manufacturer’s instructions. Quantity and quality of the total RNA were determined by NanoDrop 1000 spectrophotometer (Thermo Fisher Scientific Inc., United States) and 1% non-denaturing agarose gel electrophoresis, respectively. Strand-specific RNA-Seq libraries were generated following the method of Zhong et al. (2011). A total of 54 samples were involved in the initial experimental design. However, we found that some samples were not relevant and supportive to the research objectives and were not involved in the subsequent study. A total of 26 samples were used in this research finally.

### Identification of Differentially Expressed Genes and the Function Analysis

A total of 26 libraries derived from flesh samples were sequenced on an Illumina HiSeq 2500 with 100-bp single-end mode, and the generated datasets were uploaded to NCBI Sequence Read Archive (SRA) under the accession number PRJNA549006. Three biological replicates were carried out for each sample. The following steps were carried out to identify the DEGs. Trimmomatic was used to process the raw RNA-Seq reads to eliminate adapter and low-quality sequences (Bolger et al., 2014). Bowtie was used to align the high-quality reads to ribosome RNA database, allowing up to three mismatches. Those reads aligned to ribosome RNA sequences were eliminated (Langmead et al., 2009). Tophat was used to align the cleaned reads to watermelon genome, allowing one segment mismatch (Trapnell et al., 2009). The number of the reads mapped to each gene model of watermelon genome was counted, then normalized to reads per kilobase of exon model per million mapped reads (RPKM). To identify the genes differentially expressed in watermelon fruit flesh tissues, the getVarianceStabilizedData module in DESeq was used to transform the raw counts (Anders and Huber, 2010). LIMMA was used to analyze the transformed expression data, and F tests were carried out (Smyth, 2004). Benjamini–Hochberg procedure was used to adjust the raw *P*-values for multiple testing (Benjamini and Hochberg, 1995). GO:TermFinder was used to perform Gene Ontology (GO) term enrichment analysis of the DEGs, with the adjusted *p*-values less than 0.01 (Boyle et al., 2004). Significantly changed pathways were identified using the pathway enrichment analysis module of CuGenDB (Zheng et al., 2019). Co-expression modules and gene networks were constructed using weighted gene co-expression network analysis (WGCNA) (Zhang and Horvath, 2005).

## Results and Discussion

### Pumpkin Grafting Cultivation Extended the Period of Watermelon Fruit Development

**FIGURE 1:**
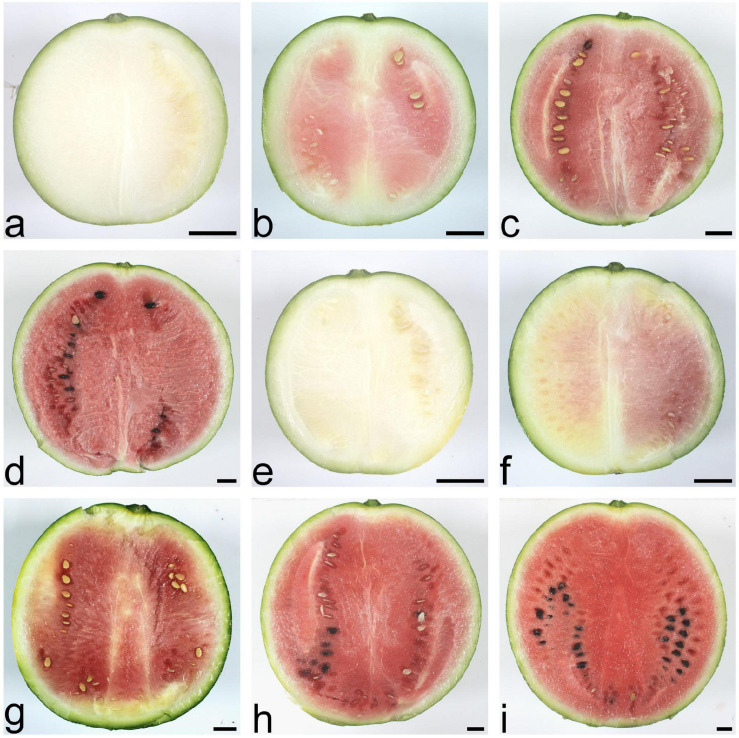
Fruit phenotypes of the watermelon self-grafted (SG) plant and the pumpkin-grafted (PG) plant at different development stages. **(a)** SG fruit at 10 days after pollination (DAP). **(b)** SG fruit at 18 DAP. **(c)** SG fruit at 26 DAP. **(d)** SG fruit at 34 DAP. **(e)** PG fruit at 10 DAP. **(f)** PG fruit at 18 DAP. **(g)** PG fruit at 26 DAP. **(h)** PG fruit at 34 DAP. **(i)** PG fruit at 40 DAP. Scale bar, 2 cm.

The scion, watermelon cultivar 97103, is a cultivated variety with high-quality fruit, round shape, moderate size, green rinds, and thin skin with light red flesh. Four important phases were involved in watermelon fruit development: immature white flesh stage (10 DAP), white-pink flesh stage (18 DAP), red flesh stage (26 DAP), and full ripe stage (34 DAP). During watermelon fruit development, the fruit traits of watermelon SG plant and PG plant changed distinctly, such as fruit flesh color and fruit size (Figure 1).

**FIGURE 2:**
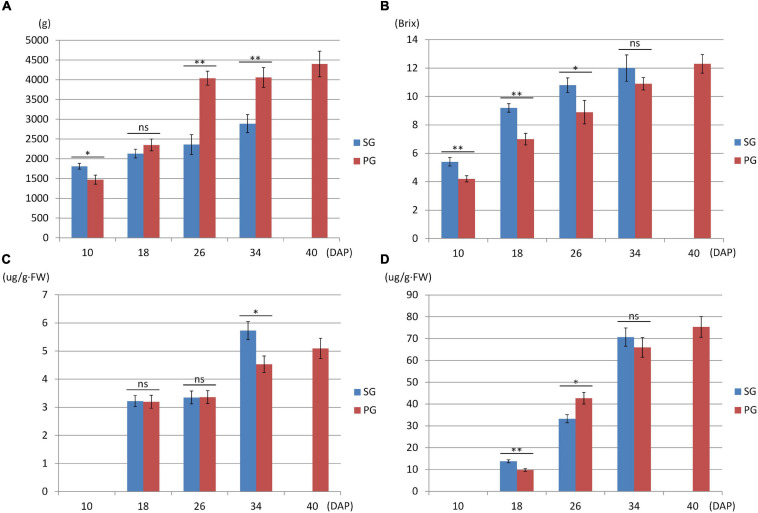
Fruit phenotype comparison between watermelon self-grafted (SG) plant and pumpkin-grafted (PG) plant. **(A)** Fruit weight. **(B)** Fruit flesh sugar content. **(C)** Fruit flesh beta-carotene content. **(D)** Fruit flesh lycopene content. ns, not significant; *P < 0.05; **P < 0.01.

Compared with the fruits of watermelon-grafted plant, the fruit development and ripening process of PG plant was extended and full ripe stage was delayed to 40 DAP. It is interesting that the quality of the full ripe PG fruit is better than that of the full ripe SG fruit, although the result is inverse in the early development stages (Figure 2). These results indicated that pumpkin grafting had a noticeable impact on watermelon fruit quality development and ripening process.

### Pumpkin Grafting Decreased Abscisic Acid Accumulation and Delayed Watermelon Fruit Ripening

Phytohormones are the important signals regulating plant growth, development, and fruit ripening. Pumpkin root was more vigorous than that of watermelon. This difference brings out the hypothesis that phytohormones may contribute to the grafting-induced impact on fruit development and ripening of watermelon scion. To test this hypothesis, five phytohormones including ABA, abscisic acid glucose ester (ABA-GE), indole-3-acetic acid (IAA), jasmonic acid (JA), and salicylic acid (SA) were determined in root and fruit of SG combination and PG combination. In comparison, we found that ABA and IAA were the major changed components among these phytohormones. In the previous study, it is found that fruit ripening was regulated by ABA biosynthesis and signaling in strawberry. In contrast, the synergic relationship between IAA biosynthesis and signaling and fruit ripening process was not observed (Guo et al., 2018). These results implied that the difference of ABA concentration may be one of the main factors leading to the delayed fruit ripening in PG watermelon. In the current study, we found that ABA concentration in SG and PG fruit flesh at 26 DAP was higher than those at 10 DAP. This result, supported by our previous study (Wang et al., 2017), indicated that ABA was the important factor promoting watermelon fruit development and ripening. Moreover, ABA concentration in PG fruit flesh was 40% lower than that of SG fruit flesh (Table 1). Fruit ripening process is regulated at multiple levels, and plant hormones such as ABA could be of great importance. In the fruits of non-climacteric type, such as cucumber, citrus, grape, and sweet cherry, ABA content was relatively low in immature fruit and increased with mature fruit and reached the peak level at the onset of ripening. The important role of ABA in non-climacteric fruit ripening is also confirmed by some mutants and exogenous treatments. ABA-deficient mutant citrus fruit caused the delayed fruit degreening process, and exogenous ABA treatment could promote fruit ripening by speeding up fruit pigment accumulation and significantly reducing the content of fruit organic acid (Romero et al., 2012). Applying exogenous ABA could significantly accelerate grape fruit ripening process, such as rapid accumulation of sugar and anthocyanin (Koyama et al., 2019). ABA application at the turning stage of cucumber fruit development could significantly reduce chlorophyll content in the exocarp (Wang et al., 2013). Injecting exogenous ABA and ABA biosynthesis promoter dimethyl sulfoxide (DMSO) could accelerate the ripening process of strawberry fruit, while application of ABA biosynthesis inhibitor nordihydroguaiaretic acid (NDGA) notably delayed the ripening process (Jia et al., 2011; Li et al., 2016). These pieces of evidence, combined with our previous study (Wang et al., 2017), suggested that the decreased ABA concentration may contribute to the grafting-induced fruit development period extension and ripening delay. This hypothesis was further supported by the result that the ABA concentration in the PG root was notably lower (98%) than that in the SG root (Table 1). ABA can be synthesized in many tissues including fruit and root. To eliminate other factors affecting fruit development and ripening, the rootstock tissue was the only different factor between SG plant and PG plant in this study. These data proved that the difference in ABA concentration between pumpkin rootstock and watermelon rootstock was the important factor contributing to fruit development period extension and ripening delay.

**TABLE 1:** Phytohormone patterns of the flesh and root tissues in watermelon self-grafted (SG) plant and pumpkin-grafted (PG) plant.

Phytohormone	DAP	SG flesh	PG flesh	Significance	SG root	PG root	Significance
ABA	10	55.23 ± 2.60	17.87 ± 0.84	***	89.79 ± 4.85	7.71 ± 0.40	***
	26	230.79 ± 13.28	143.76 ± 7.58	**	714.35 ± 37.36	15.71 ± 0.69	***
ABA-GE	10	1.24 ± 0.05	0.15 ± 0.02	***	1.69 ± 0.09	6.72 ± 0.33	***
	26	8.51 ± 0.56	3.12 ± 0.19	***	26.99 ± 1.47	5.36 ± 0.28	***
IAA	10	1.39 ± 0.07	3.2 ± 0.16	***	4.33 ± 0.25	3.1 ± 0.14	**
	26	68.58 ± 3.81	12.47 ± 0.70	***	11.91 ± 0.69	2.84 ± 0.12	***
JA	10	4.55 ± 0.28	2.16 ± 0.11	***	8.04 ± 0.45	15.68 ± 0.82	***
	26	0.49 ± 0.03	0.28 ± 0.03	**	7.47 ± 0.40	8.68 ± 0.52	ns
SA	10	34.97 ± 2.13	16.38 ± 0.85	***	245.47 ± 13.30	75.69 ± 4.27	***
	26	19.98 ± 0.89	2.03 ± 0.03	***	1,148.97 ± 68.90	41.17 ± 2.35	***

ns, not significant; **P < 0.01; ***P < 0.001. ABA, abscisic acid; ABA-GE, abscisic acid glucose ester, IAA, indole-3-acetic acid; JA, jasmonic acid; SA, salicylic acid.

### Identification of the Differentially Expressed Genes Between Pumpkin-Grafted and Self-Grafted Watermelon

**FIGURE 3:**
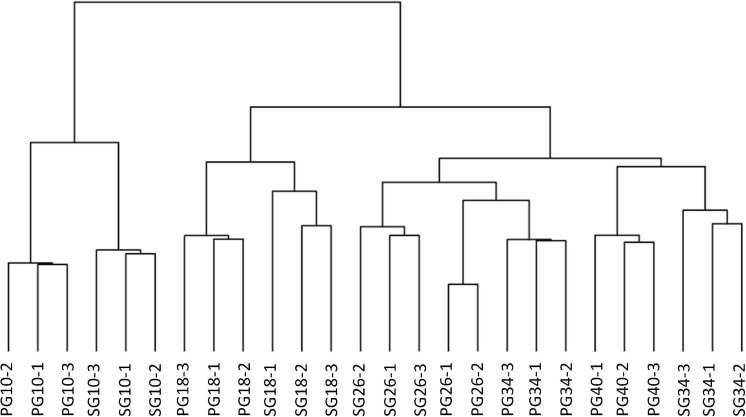
Cluster of the differentially expressed genes in fruit of self-grafted (SG) watermelon plant and pumpkin-grafted (PG) watermelon plant.

To identify the candidates contributing to grafting-induced difference in fruit development and ripening, we performed the pairwise comparison of the expressed genes at the four critical stages. There were 4,102, 1,817, 1,760, and 1,675 genes differentially expressed between SG fruit and PG fruit at 10, 18, 26, and 34 DAP, respectively. Sample clustering showed that the PG fruits at both 26 and 34 DAP were clustered with the SG fruits at 26 DAP. Similarly, PG fruits at 40 DAP were clustered with the SG fruits at 34 DAP (Figure 3). This reflects the fact that the fruit development period of the PG watermelon was extended compared with the SG watermelon. This extension may facilitate more photosynthate accumulation in fruit and promote the fruit enlargement and the quality improvement finally.

### Gene Ontology Function and Pathway Enrichment of Differentially Expressed Genes

**FIGURE 4:**
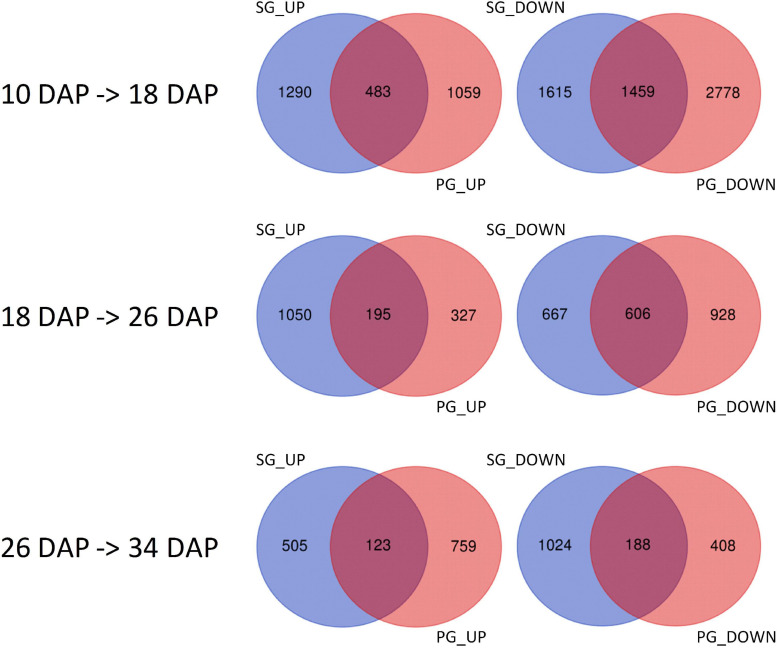
Identification of the differentially expressed genes in self-grafted (SG) watermelon plant and pumpkin-grafted (PG) watermelon plant. DAP, days after pollination; SG_UP, up-expressed genes in SG plant; SG_DN, down-expressed genes in SG plant; PG_UP, up-expressed genes in PG plant; PG_DN, down-expressed genes in PG plant.

Among these DEGs, 1,765, 1,103, 1,427, and 912 genes were downregulated in the PG fruit compared with those in the SG fruit (Figure 4). Before 26 DAP, most of the downregulated genes in the fruit of SG combination were also downregulated in the PG combination. The upregulated genes showed a big difference. Those commonly upregulated genes may play an essential role in fruit ripening. GO enrichment analysis indicated that cell development and phytohormone signal-related functions were enriched at 10 DAP, such as regulation of nitrogen compound metabolism (GO:0051171), regulation of hormone levels (GO:0010817), regulation of cellular biosynthetic process (GO:0031326), cellular hormone metabolic process (GO:0034754), cytokinin metabolic process (GO:0009690), cellulose biosynthetic process (GO:0030244), cellular response to hormone stimulus (GO:0032870), and ABA binding (GO:0010427). These DEGs enriched in these gene function categories may contribute to the fruit development delay in the early stage and the relatively smaller fruit (1.47 kg) of the PG combination than that of the SG combination (1.81 kg) in the early fruit development stage. At 18 DAP, the functions of these DEGs were mainly enriched in carbohydrate metabolic process-related categories, such as monosaccharide metabolic process (GO:0005996), negative regulation of cellular biosynthesis process (GO:0031327), hexose metabolic process (GO:0019318), carbohydrate metabolic process (GO:0005975), and carbon fixation (GO:0015977).

**FIGURE 5:**
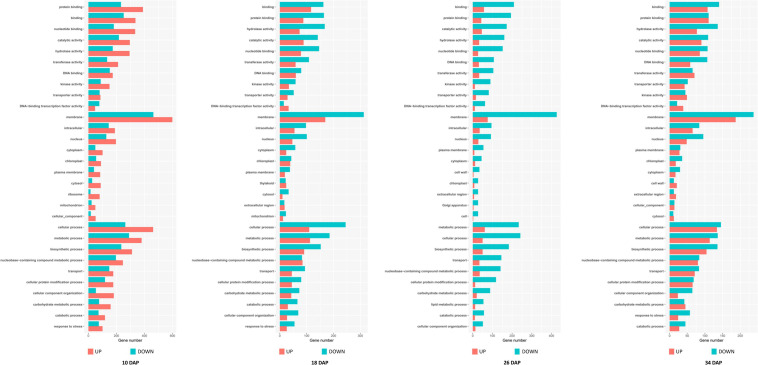
Gene Ontology (GO) terms enrichment of the differentially expressed genes in self-grafted (SG) watermelon plant and pumpkin-grafted (PG) watermelon plant.

Downregulation of these DEGs may contribute to the decreased fruit sweetness in fruit (7.2 Brix) of the PG combination compared to the fruit of the SG combination (9.0 Brix). The downregulated DEGs at 26 DAP were mainly enriched in phytohormone regulation, fruit sweetness, and flavor and texture development-related function categories, such as hormone transport (GO:0009914), carbohydrate metabolic process (GO:0005975), organic acid transmembrane transport (GO:1903825), galactose metabolic process (GO:0006012), xyloglucan metabolic process (GO:0010411), hemicellulose metabolic process (GO:0010410), regulation of hormone levels (GO:0010817), cell wall polysaccharide metabolic process (GO:0010383), polysaccharide metabolic process (GO:0005976), cell wall biogenesis (GO:0042546), and cellulose metabolic process (GO:0030243). Normally, watermelon became ripe at 26 DAP (Figure 5). In the PG watermelon fruit, several fruit quality traits are significantly lower than those of the SG fruit, including fruit sweetness, flesh color, and flesh texture, except fruit size (Figure 2). These results suggested that downregulation of these phytohormone regulation and fruit quality-related DEGs in the PG combination were important factors delaying watermelon fruit ripening. Similar to the fruit morphological difference at 26 DAP, the fruit qualities, including fruit sweetness, fruit beta-carotene content, and lycopene content, of the PG combination were still lower than those of the SG combination at 34 DAP (Figures 1, 2). At this stage, functions of these DEGs mainly enriched in fruit nutrition components and ripening-related categories included lipoprotein catabolic process (GO:0042159), cysteine biosynthetic process (GO:0019344), fruit ripening (GO:0009835), and developmental maturation (GO:0021700).

Compared to the SG combination, another notable fruit trait in the PG combination is that the fruit size was enlarged after 10 DAP stage. GO enrichment analysis of the upregulated DEGs indicated that several nitrate metabolism and cell wall development-related categories were enriched, including cell wall organization (GO:0071555), nitrate metabolic process (GO:0042126), nitrate assimilation (GO:0042128), xylem development (GO:0010089), and negative regulation of cell death (GO:0060548). The involved DEGs may contribute to the pumpkin grafting-induced fruit development period extension and fruit enlargement. This result preliminary explained the mechanism of grafting-induced difference of watermelon fruit development.

### Expression of Abscisic Acid Centric Signal Regulators Were Altered in Pumpkin-Grafted Watermelon

To understand the co-expression dynamics during watermelon fruit developmental process between the two different cultural methods and to identify the pivotal genes highly associated with grafting, WGCNA was carried out and 24 distinct modules were identified. The co-expression modules were associated with the fruit development stages. The modules that highly correlated with pumpkin grafting cultivation and displayed the opposite correlation with self-grafting cultivation were analyzed further to investigate the gene regulatory network affected by grafting.

**FIGURE 6:**
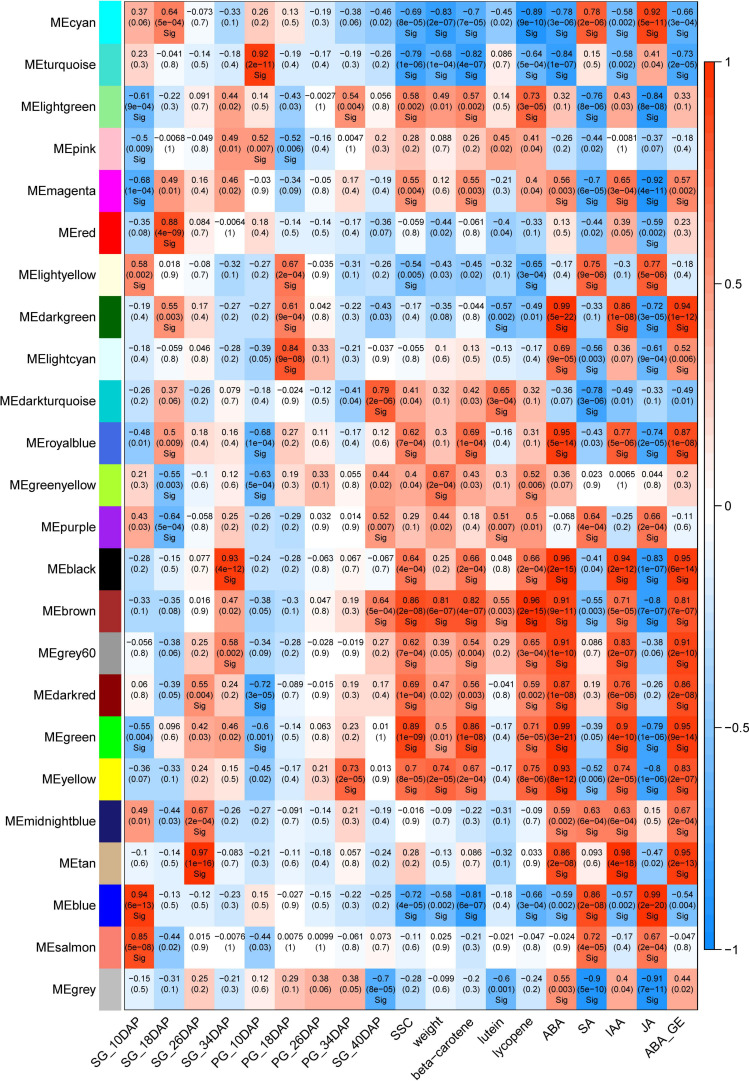
Weighted gene co-expression network analysis (WGCNA) module analysis of the differentially expressed genes in self-grafted (SG) watermelon plant and pumpkin-grafted (PG) watermelon plant.

Two modules, tan and darkturquoise, were identified to be highly positively correlated to the 26 DAP SG fruit and 40 DAP PG fruit, respectively (Figure 6). Moreover, we identified that the brown module is highly correlated with all of the fruit quality traits including soluble sugar content (SSC), weight, beta-carotene, lutein, and lycopene, and fruit development and ripening-related phytohormones (ABA, IAA, ABA-GE). It is also highly correlated with the PG fruit at 40 DAP, the important stages with improved fruity quality and size than the SG fruit at 34 DAP. Sample clustering indicated that the PG fruit at 40 DAP was clustered with the SG fruit at 34 DAP. The fruit size, fruit sweetness, and fruit color of the PG fruit at 40 DAP are better than those of the SG fruit at 34 DAP. In our previous study, we found that ABA content in fruit flesh is significantly correlated with watermelon fruit ripening and fruit quality evolution. Significant correlation (*R*^2^ = 0.905) was observed between ABA content and SSC in ripening watermelon fruits of different evolutionary stages (Wang et al., 2017). These findings, combined with our previous study, suggested that these genes in the brown module may be the important candidates contributing to the grafting-induced watermelon fruit size enlargement and fruit quality improvement.

**FIGURE 7:**
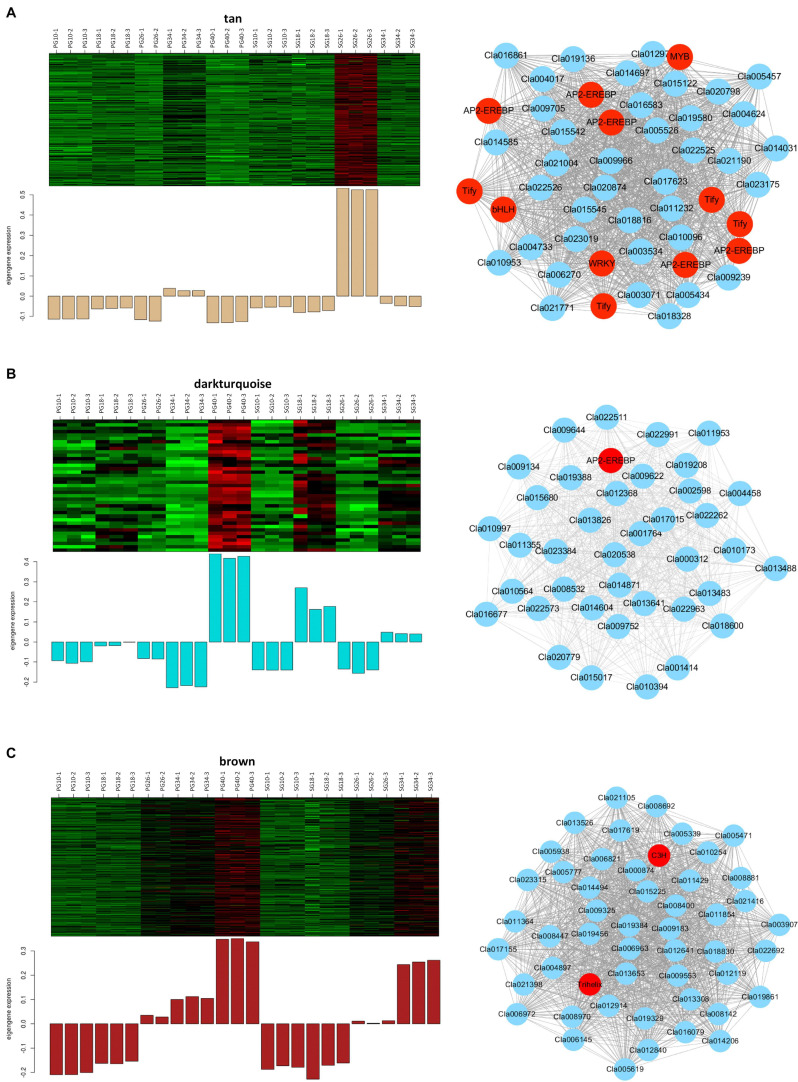
Expression patterns and the co-expression networks of the differentially expressed genes in self-grafted (SG) watermelon plant and pumpkin-grafted (PG) watermelon plant. **(A)** The module correlated with SG fruit at 26 days after pollination (DAP). **(B)** Darkturquoise module correlated with PG fruit at 40 DAP. **(C)** Brown module correlated with PG fruit at 40 DAP.

Considering that these modules might contribute to the fruit quality difference between the SG plant and the PG plant, the gene networks of these modules were analyzed further to identify the vital genes. A total of 232 genes were involved in the tan module that significantly correlated with the SG fruit at 26 DAP (Figure 7A). Co-expression networks indicated that five AP2-EREBP transcription factors (Cla016009, Cla002330, Cla021070, Cla021069, Cla011487), four Tify transcription factors (Cla011143, Cla019575, Cla009781, Cla012536), one bHLH transcription factor (Cla007559), one MYB transcription factor (Cla009156), and one WRKY transcription factor (Cla015003) served as the hub gene of this module. These hub genes represented the core nodes of this co-expression network. They may determine the difference between the SG fruit and the PG fruit at 26 DAP by regulating other or phytohormone signaling and fruit quality-related genes involved in this module, such as PP2C proteins (Cla009239, Cla016081, Cla013306, Cla020413, Cla009546) and trehalose 6-phosphate phosphatases (Cla006270 and Cla008123).

A total of 39 genes were involved in the darkturquoise module that significantly correlated with the PG fruit at 40 DAP (Figure 7B). Co-expression networks indicated that an AP2-EREBP transcription factor (Cla020392) served as the hub gene of this module. AP2/EREBP transcription factor family is a plant-specific transcription factor, which plays vital and multiple roles in plant growth, development, and fruit ripening. This result suggested that the AP2-EREBP transcription factor-regulated gene network contributes to the grafting-induced fruit quality improvement.

The brown module is highly correlated with all of the fruit quality traits (SSC, weight, beta-carotene, lutein, and lycopene) and fruit development and ripening-related phytohormones (ABA, IAA, ABA-GE). It is also highly correlated with the SG fruit at 40 DAP (Figure 7C). A total of 1,557 genes were involved in this module. Co-expression networks indicated that the trihelix transcription factor GT-3b Cla017995 and the C3H transcription factor Cla018368 served as the hub genes. Besides the two transcription factors, there were 80 transcription factors that were involved in this module, including NAC transcription factor Cla019693, bHLH transcription factor Cla022045, and MADS transcription factor Cla014493. Moreover, several ABA signaling and fruit quality-related candidates were involved in this module, such as ABA receptor PYR1 Cla006604, sucrose transporter Cla005565, and carotenoid isomerase Cla011810. These ABA signaling and fruit quality-related genes may be the important candidates determining the difference between the SG fruit and the PG fruit.

## Conclusion

Watermelon, the typical non-climacteric fruit plant exhibiting several unique characteristics, has attracted numerous studies to explore the intrinsic mechanisms of the fleshy fruit development and ripening. Grafting became a widely used cultural method in watermelon production, considering the serious outbreaks of soilborne disease in watermelon production. The morphological traits of watermelon fruit including fruit size, flesh sweetness, color, and texture were changed dramatically by different grafting cultural methods. The impact and intrinsic mechanism of grafting-induced watermelon fruit quality improvement were not well-elucidated. In our study, the fruit flesh and root tissues from critically different fruit development periods of watermelon SG plant and PG plant were analyzed. We found that the fruits of SG and PG dramatically changed in morphological traits, such as fruit sweetness, flesh color, and fruit weight. Self-grafting in the same developmental stage increased fruit flesh sweetness, while pumpkin grafting decreased flesh sweetness. Compared with the fruits of the watermelon-grafted plant, fruit development, and ripening process of the PG plant were extended and full ripe stage was delayed to 40 DAP. It is interesting that the quality of the full ripe PG fruit is better than that of the full ripe SG fruit, although the result is inverse in the early development stages. Phytohormone dynamics suggested that the difference in ABA accumulation was the main factor leading to delayed fruit ripening of grafted watermelon. Further transcriptome analysis indicated that ABA signal regulators were differentially expressed and played key roles in the fruit ripening gene expression network of grafting watermelon (Supplementary Table S1). This study provides a systematic insight on the difference between SG watermelon and PG watermelon in fruit metabolites, phytohormone, and gene expression levels for the first time. Our findings of ABA centric phytohormone difference-mediated fruit ripening delay in PG watermelon will help to get the comprehensive understanding of grafting plant development mechanism and improve the rootstocks beneficial for fruit development and quality characteristics.

## Data Availability Statement

The datasets generated for this study can be found in the online repositories. The names of the repository/repositories and accession number(s) can be found below: https://www.ncbi.nlm.nih.gov/, PRJNA549006.

## Author Contributions

YX, HL, and SG conceived the research. YX and SG designed the experiments. SG, GZ, GG, YR, JZ, ML, and HZ performed the experiments. SG, HS, and JT analyzed the results and wrote the manuscript. All authors approved the final manuscript and agreed to be accountable for all aspects of the work in ensuring that questions related to the accuracy or integrity of any part of the work are appropriately investigated and resolved.

## Conflict of Interest

The authors declare that the research was conducted in the absence of any commercial or financial relationships that could be construed as a potential conflict of interest.
